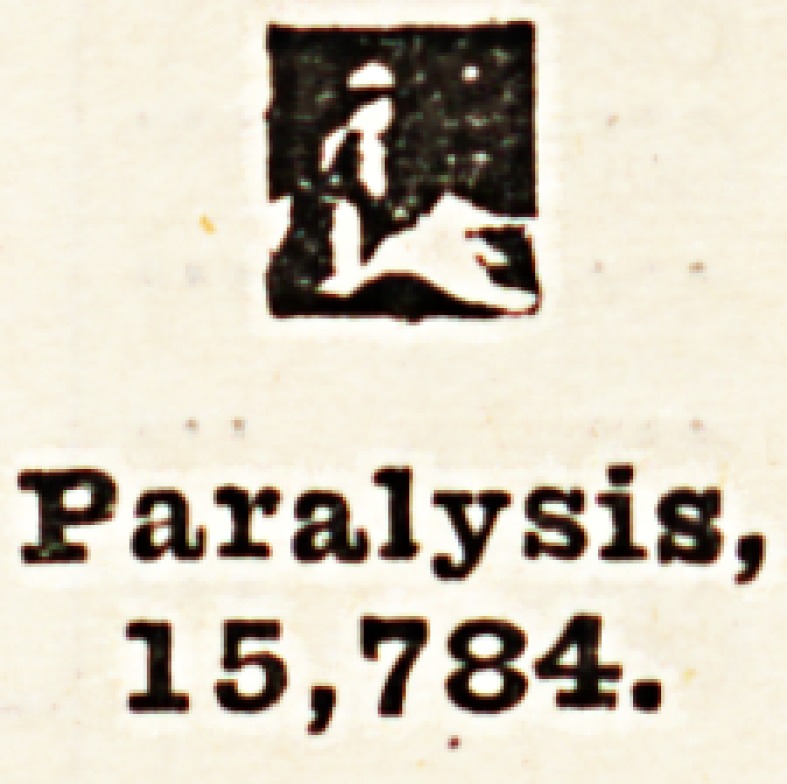# Special Hospital Sunday Supplement

**Published:** 1898-06-11

**Authors:** 


### Special Hospital Sunday Supplement.

### HOSPITAL SUNDAY IN LONDON.

### THE CHARITY OF IT.

The time has come round once more when it is our privilege to state the case in favour of a larger volume of contributions to the hospitals through the congregational collections in the metropolis on Hospital Sunday. It may be well at the outset to remove some misapprehension by pointing out that the Prince of Wales's Hospital Fund is in a fair way to secure the object for which it was instituted, namely, the provision of an income for the voluntary hospitals equal to the existing annual deficiency beyond the total available resources derived through the ordinary channels. Still the necessity for the Hospital Sunday collections is as pressing as ever. This is so because Hospital Sunday is one of the ordinary sources of revenue for the voluntary hospitals of London, and the Prince of Wales's Fund, however successful, cannot properly fulfil its objects should the collections on Hospital Sunday in the aggregate amount to much less than £60,000 per annum. Before the institution of the Prince of Wales's Fund many preachers, and many givers, too, were discouraged by the feeling that, do what they could, it was impossible for them to wipe out the annual deficiency of the hospitals. Now that the institution of the Prince of Wales's Hospital Fund has removed this cause for despondency, we hope and believe that the Hospital Sunday Fund will produce on an average at least £50,000 per annum.

The Daily Round.—Out Patients Waiting for Medicine.

Callous people and sceptics have maintained that there is no charity in subscribing to hospitals, seeing that hospitals must exist, and that, if they are not supported by the free offerings of a free people, Parliament must enact that the hospitals shall be set up and maintained at the expense of the ratepayers. We have been surprised that any minister of religion should entertain any such idea as this for a single instant. Of all the parables, that of the Good Samaritan is probably the one which has most appealed to public sentiment, and has thus led to the enforcement of Christian principles which otherwise might have failed to convince the multitude. Works of mercy have always been, and are still, the most certain means of raising the character of those who engage in them. Ministers of religion know that any mere expression of religious feeling, unless it bears practical fruit in good works, is very often more dangerous to the individual than an attitude of indifference or ignorance. In the latter state a man may always be open to conviction, whereas in the former he is apt to beget a self-satisfied confidence which may produce more evil than good. Therefore we make bold to say the greater the experience of the teacher and preacher, the stronger will be his conviction that it is of the first importance to the nation that the principle of voluntary offerings for the maintenance and care of the sick should be preserved through the direct instrumentality of all ministers of Christ.

The true charity of giving in support of hospitals is surely self-evident. Every man of business who has time and power for thought must recognise the economy to Londoners of promoting the success and efficiency of the great voluntary hospitals. It is bad economy to keep the bread-winner of the family away from his work, for everyone knows that every day that he is away, and unable to labour and to earn his pay, the powers of recuperation of his family grow less and less. It is bad economy, too, to let children grow up maimed by injury or dwarfed by disease, when a reasonable contribution to the hospitals will supply the means of making such children strong and useful members of the community. The poor have ever been regarded by right thinking people, who have means, as their especial care; but the sick poor, those members of the artizan and humbler classes who depend upon their daily work for their daily bread, and without whose constant labour a great city like London would be well nigh uninhabitable, are especially in the keeping of all who have it in their power to give. Without hospitals the poor must suffer and lose their power to work which would mean loss and discomfort to the whole community.

The patience of these poor people under suffering is as remarkable as it is encouraging. When people talk of the abuse of the hospitals they very often have not the smallest apprehension of the ills endured, with uncomplaining patience, by the patients, who consist mainly of men and women that are the mainstay of their families, and whose illness is the cause of great affliction to others and especially to children. Such a bread-winner in a hospital, however much he or she may suffer physically, must often endure hours of mental anxiety, amounting in many cases to acute pain, owing to the knowledge that every day he remains in the hospital bed the family at home are approaching nearer and nearer to the time when there will be neither bread to eat nor clothes to wear.

It is ignorance which produces indifference in the majority of cases. Many persons persuade themselves that they are clever because they decline to recognise the privilege of giving to the support of hospitals. This being so, Hospital Sunday affords every preacher an opportunity of attacking this indifference and enlightening such ignorance by means of the lessons which the hospitals teach and which the preacher can drive home to the consciences and mind of his hearers. Every year an increasing number of ministers of religion recognise Hospital Sunday as an occasion to be welcomed. It gives them an occasion to enforce principles of love and charity, making them fruitful for good to the members of the congregation. It is true, of course, as ministers of religion are but men, that amongst the whole number there must necessarily be some who either lack the intelligence, or the knowledge, or the sympathy to realise what a grand text Hospital Sunday is when the preacher has the wisdom to avail himself of it, and to take pains to get up his subject properly. Throughout London there are many places of worship which are almost identical so far as the character and resources of the congregation are concerned. Any minister of religion who is a good citizen must be perfectly aware of this fact, and perfectly able to make out a list of congregations which are identical in character with his own. In this way, by obtaining a report of the Hospital Sunday Fund Council he will be able to see how far his own people are doing their part, when compared with other congregations, in the good work of supporting the hospitals. This is the course which was originally pursued by Canon Miller, the founder of Hospital Sunday, who was probably more successful than anybody else in quickening the sympathies of the hale in favour of the sick.

The hospitals are ever ready to provide for the sick poor who are known to the clergy and ministers of religion who make collections on Hospital Sunday. It follows that preachers must be aware of the great practical value of the Hospital Sunday Fund to the poor of their parishes and congregations, seeing that it enables the parishioners to obtain without cost adequate medical appliances in addition to hospital and convalescent aid. On all these grounds the charity of Hospital Sunday becomes plain to all who will give a little time to the consideration of the subject. Everybody can give liberally and heartily to the support of hospitals on Hospital Sunday, knowing that by so doing they secure a guarantee that their contributions will be expended upon an object worthy of the support of every intelligent citizen in the metropolis of the Empire.

ADMINISTERING CHLOROFORM.

### BLESSED CHLOROFORM.

In the daily round comes chloroform. What an untold blessing is this! Without it what excruciating pain must be endured hourly in the great hospitals and sick chambers of the wide world. The anæsthetist is a responsible officer. To him the case upon which he is now engaged may be only one of twenty or thirty during the day, but the greatest care and the most delicate judgment must be separately exercised. As a rule, hospital patients, whose cases necessitate an operation, are eager for the arrival of the day or the hour when they shall go to the theatre; they date the turn in their time of suffering from the event. Patient number six talks to his or her neighbours in beds number five and seven on the subject, and often, as the Sister approaches the bedside, may be heard to say, "Well, I hope the performance in the theatre will be a success." But this would not be the case but for the knowledge that chloroform, or some anæsthetic, will take away all feeling during the operation. This, again, is part of the daily round, one of the blessings offered to the poorest by the hospitals dotted all over the metropolis.

### THE NECESSITY FOR HOSPITALS IN A GREAT CITY.

### I.—FROM A POLITICAL POINT OF VIEW.

To be free a people must be self-contained, independent, and careful to provide a system of government which meets adequately all the needs of every class of the community. No Government is politically sound which fails to recognise the imperative necessity of securing the greatest good for the greatest number. The humbler citizens must be made to realise that those more prosperous than themselves, those who control the Government, who possess the means to provide adequate comfort for their own families, are manifestly solicitous for the welfare, health, and happiness of every member of the community. Sickness is common to all classes and each individual, but its burden is very unequally distributed, so far as its effects upon the family of the rich and the poor are concerned. The rich, as a matter of course, are well able to obtain every comfort in sickness, and every advantage which the highest medical skill and the best nursing can afford them. Without hospitals of the utmost efficiency the poor must suffer much preventable misery in the day of sickness. It is the privilege and duty of the rich and well-to-do to provide adequate funds to enable ample provision to be made whereby the poor, when overtaken by accident or when suffering from disease, may be provided with everything which can hasten their recovery and re-establish their physical powers so as to enable them to maintain their families. There cannot be any doubt that, as the world grows richer, it is apt to grow more selfish also. The great mass of the well-to-do fail to realise the duty and privilege of giving to hospitals regularly and in proportion to their income and resources. They are bound to do this as Christian men and women, and the world would be infinitely worse than it is at present if none but State-supported hospitals were maintained for the succour of the sick. The effect of State support upon all classes of the community must tend to widen the distinction between the classes, to harden the relations which exist between the rich and the poor, because the privilege of giving, when appreciated as it ought to be by those who have the power to give, promotes tenderness, and affords opportunity for that touch of nature which makes the whole world kin. All honour then to that increasing number of the well-to-do who give liberally of their substance to the succour of the sick. But those men and women who do not realise the duty and privilege of giving to the support of hospitals must be in reality indifferent citizens, with a tendency to lapse into luxury and selfishness at the expense of the higher attributes of manhood. These attributes of manhood are the backbone of nations, and history proves that as those attributes are lost sight of, and where they fail to be successfully cultivated, there nations degenerate more and more, until they become effete. Again, on the mere ground of selfishness, it is highly desirable that those who have it in their power to give should give cheerfully to the hospitals, for at the hospitals medical men and nurses are trained, and without efficient medical and nursing service the lot of rich and poor alike in illness would indeed be unhappy.

THE DAILY ROUND.—CROUP.

As a matter of economy State-supported hospitals are a mistake. Those who know most about the history of these State institutions, all over the world, are aware that they tend to be less efficient and more extravagant than any other class of institution. Abuses creep in like those which were exposed in connection with the Eastern Hospital scandals, which interfere with the efficiency of the medical treatment, the comfort and recovery of the patients, and the well-being of all who are connected with them. Rate-supported institutions tend to pass out of public notice, and small interest is taken in their efficiency and condition, because what is everybody's business in practice becomes nobody's concern. Further, State-supported institutions do not progress with the times. They have never encouraged the development of the highest scientific discoveries, nor have they ministered to scientific progress or to improvement in the methods adopted for the care and cure of the sick.

Finally, we are convinced that Englishmen of the present generation would feel it a reflection upon their manhood if they were to permit the voluntary hospitals of this country, which they owe to their forebears and sires, to be abolished in favour of State supported hospitals. The necessity for any such change can only arise if and when the sense of public duty, the sense of the individual responsibility of man to man, becomes less and less recognised, and so evidence were supplied of the degeneracy of the race. Of course there are a number of people, who if they are not thoughtless must be deliberately callous, who contend that all our hospitals ought to be maintained out of the rates. We have taken some trouble to examine the records of the men who have been foremost in the advocacy of this system, and we have come to the conclusion that they belong to the type of non-givers. It is so simple to advocate rate support as a panacea for everything. This eternal demand for State control and State aid is in itself proof of the degeneracy of those who make it. They thus exhibit a loss of manliness and self-respect which proves them to be feeble folk whom no man can properly follow. We repeat properly follow, because such advocates show an ignorance of the facts of taxation and its burden upon the people which rules them out of Court. If all the hospitals were to be rate-supported, Londoners would have an addition to their taxation which would drive most people out of the metropolis who could afford to reside elsewhere. Those who were left might thus find the rates so heavy as to be ruinous. In any case, whatever the original amount of the hospital rate, it would tend to increase largely year by year. If the voluntary hospitals are restricted to at present by a few of those able to pay for treatment, then everybody would go to the hospitals when sick as a matter of right, because these institutions were entirely maintained by taxation. Thus, the last state would be worse than anything conceivable under our present system, which places wholesome restrictions on free relief, maintains the independence of the citizens, conserves true charity, secures scientific progress, and cultivates reciprocal and kindly feelings between man and man, whereby the whole race is built up and continuously improved. The British public will, we are convinced, prefer to support and uphold the work of the worthy men and women who devote their lives to the maintenance of the voluntary hospitals and their efficient management. It is so placed beyond doubt that most sensible persons think that State-supported hospitals would be an unmixed evil. Indeed, no one worthy of his day and generation could conscientiously support their establishment in place of the great voluntary hospitals, which are properly regarded as amongst the most glorious institutions of the British Empire.

THE DAILY ROUND.—UP TO THE WARDS.

It has been well said that "if a man cannot look down with tenderness he cannot look up with hope." So long, then, as Christianity is a vital force in the national life of England, so long may we rest certain that the voluntary system of hospital support will prevail throughout the British Empire by the deliberate will and decision of the people of England.

We have spent many hours on several occasions in the out-patient departments of the great voluntary hospitals of this country. We have made it our business to carefully inquire into the cases, their urgency and character. We have studied the circumstances of the people who attend the casualty departments, and we have come to the conclusion that should the day ever arrive when the British people decline to maintain the voluntary hospitals, on that day England would cease to be free in any proper sense, and the day of anarchy and upheaval would have come. Apart altogether from sentiment, no thinking man, no statesman, who is possessed of a knowledge of the facts, can have the slightest doubt that to abrogate the principle of the voluntary support of the great hospitals of England would amount, in fact, to a denial by the well-to-do classes of the rights and claims of the poor, and would afford startling evidence of degeneracy in the race, and prove the beginning of a war between the classes which must ultimately end in the confusion of all government, the commencement of civil strife, and the eventual decay and destruction of the Empire. Thoughtless people are harmless so long as they are inactive, but when they persistently formulate proposals like that for the substitution of State support for voluntary support in regard to hospitals, they may degenerate into a dangerous nuisance, and should be promptly suppressed by the thinking portion of the community.

### II.—FROM A MEDICAL POINT OF VIEW.

IN view of the great annual collection which will shortly be made for the Hospital Sunday Fund, it is important that every misapprehension on the subject of hospital administration should be removed, and there is one misapprehension which it is especially desirable to clear away, namely, the idea that medical men as a class are opposed to hospitals. The idea is quite a false one. No class probably admits more fully in its inmost heart than do medical men that the work of the hospitals is good, that it is work which cannot otherwise be done, and that so far as anything at all can be spoken of as necessary, the hospitals are an absolute necessity in the present state of society. Yet if one takes up the medical papers one often finds grumblings against the hospitals, and there is no doubt that it is to these that is due the impression to which we have referred. But it must be remembered that the medical papers are technical journals in a double sense, for they voice the profession not merely in regard to science, but also in regard to what we may call the bread-and-butter side of the doctor's life, and thus we find in them displayed all the irritations and annoyances to which the general practitioner is subjected in consequence of the competition to which he is exposed on every side. Perhaps, then, it is not to be wondered at that the more loudly the hospitals proclaim the extent of the good they are doing, as measured by the multitude of patients they relieve, the more the doctors cry out about hospital abuse. It is but natural. There is no doubt that in all large towns there are more medical men than can find full employment, some of whom are in dire distress, and that to such the tales which are current about well-to-do hospital patients, tales which are to be heard wherever two or three medical men are gathered together, are as gall and wormwood. So long as charity continues — and long may that be—we may be sure that some unworthy hands will always be held out.

The Daily Round.—A Painful Spot, but "Doesn't Mind."

That these unworthy recipients of hospital relief are not so numerous as some would have us believe, and that their elimination is not so simple a matter as some promoters of schemes for hospital reform seem to imagine, is, we think, shown by the fact that the outcry among medical men is as old as the hills, and that it remains just as loud as ever it was, notwithstanding that the remedy of the abuse, so far as it exists, lies to a large extent in the hands of the medical men themselves. How many commissions, how many committees have there not been on this very subject; how many associations have not been formed by medical men to promote reform, how many conferences have not been held; and yet with what result? Practically nothing.

Notwithstanding all the talk of all these years, and notwithstanding the fact that the medical profession is an absolutely close corporation, and moreover that it possesses a powerful, wealthy, and representative association, which includes in its membership the staff of practically every hospital in the kingdom—notwithstanding all this, the things of which the medical profession is said to complain continue to go on under its very nose, and with the concurrence and assistance of its most noted leaders. We think that inference is fair that the complaints which are from time to time made by doctors against the hospitals are not so real as some would have us believe, and that so far as the public, and especially the giving public, are concerned, they may be put on one side until the medical profession offers something like an authoritative pronouncement as to the proper treatment to be applied to them. Till that is done certainly the idea that medical men at large are opposed to hospitals must be taken as erroneous, and must on no account be held as a valid excuse for the withholding a single sixpence from the Hospital Sunday Fund. For, indeed, whatever medical men may say in a loose sort of way about hospitals at large there is one hospital which in the eyes of each of them can do no wrong, one hospital which he regards with pride and with affection, namely, his *alma mater*, the hospital where he was educated, the one whose very name recalls the scenes of his youth, the student years, the growth of knowledge, the friendships which have lasted through his life. In regard to that hospital, which is the one he knows most about, a doctor will generally refuse to listen to a word of reproach.

It is not, however, merely as the places of their education, and as names recalling early days before life's disillusions came upon them, that medical men look upon the great hospitals with affection and respect, but also because they remain to them throughout their lives the fount of knowledge. No one can watch the progress of medical science without becoming aware that it is to the hospitals, and to the work done in them, that most of it is due. The general practitioner, when in doubt and anxiety about his cases, does not refer to the writings of another general practitioner, if, indeed, such exist, but to the published experiences of the hospitals. It is, in fact, a matter of no small interest to observe how petty are the contributions to medical science made by the enormous mass of isolated general practitioners, compared with those which come from the investigations carried out in a more or less co-operative manner by the comparatively small body of physicians and surgeons attached to hospitals. We would not speak lightly of the skill of the private practitioner. In his daily work he piles up for himself an enormous store of that precious thing experience, which is of immense assistance both to himself and to his patients. But the experience so gathered, however valuable, is apt to be personal, and peculiar to himself, and of small value to the world. By dint of experience a man may arrive at such a point that with his eyes shut he can diagnose a disease by smell alone. An admirable faculty, no doubt; but who can teach a smell? and what is the world better for experience which dies with the individual?

Far different is it with hospitals, and especially with those great hospitals election to the staff of which is not only an honour, but an entry into a society of good-fellowship and common work, within which knowledge gained by one is shared in by the whole, and in which all members work together not merely for the advancement of science, but for the credit of the school. It is the shortness of life and the limitations placed upon individual experience which render hospitals so essential for the advance of medical science. A strange case which one man sees once in a lifetime is no use to anyone; but such a case in a hospital gets upon the records, such a case is shown to other members of the staff, it is compared with the records of years gone by, and ultimately it finds its place in publications open to the world. We repeat, the staff of a great hospital does not consist of a number of individuals each working for self, but of a mass of men working together for common objects; and it is this fellowship, this mutual help, this discussion of cases, this showing to all of anything peculiar in the practice of one, it is by the publicity of all that is done, by the maintenance year after year of records of all that is seen, by the development of comradeship in work, and even by the growth of *esprit de corps* and sound traditions, that hospitals are able to advance medical science in a way that would be absolutely impossible without them.

THE DAILY ROUND.—PRACTISING ON EACH OTHER. A NURSES' CLASS.

The relation of the hospitals to medical science is not merely that they are places of medical education, but that they form the ever-flowing fount from which medical knowledge proceeds for the guidance of the practitioner in whatever part of the world he may be placed. It is on the knowledge so obtained in hospitals, knowledge which could not be obtained in any other way or by any other form of institution, that the lives of men and women in all parts of the world depend. When people, then, are asked to support the hospitals, let them not think that they are asked to give what to them personally is unlikely to bring any return. This is a selfish view, no doubt, but it is worth considering; for it must be insisted on that when disease comes, when sickness creeps on, when suffering has to be borne, and when help is called] for, as it is called for insistently every day from medical science, it is from the hospitals that that help comes. The practitioner is a good man, he is a kindly friend; his is the hand that gives comfort and assuages pain. In doing all this, however, he is but acting as a medium. He gives comfort, he cures disease, and he relieves pain, and for so doing let us pay him well; but if we search back to the beginnings of things we find that these benefits which the rich enjoy, and the provision of which they demand so earnestly when ill, comes primarily from the hospitals. The hospitals, then, do not benefit those alone who lie within their walls; the good they do, a form of good which could not be done in any other way, spreads over the whole world, and those who give to their support while giving to the poor give also to themselves.

### III.—FROM A SOCIAL POINT OF VIEW.

In the good old days, when intention counted for more than result, it would hardly have been necessary to offer any justification for the existence of hospitals. The pious founder or contributor helped the poor, and the infirm to boot, and thereby saved his own soul. But now we are asked, not if the founder meant well, but if he did well; if the poor were really helped in such fashion as they could not have attained for themselves; or, if instead, the lazy and dishonest were enabled to escape a burden which they could and ought to have borne. The fierce light of criticism has beat upon our hospitals, and, therefore, it will be well to state once more the reasons for which we hold that the voluntary hospitals are not only desirable but necessary institutions.

Hospitals exist for two reasons—the cure of disease in such cases as cannot pay for medical help, and the training of young men as doctors. The first of these may be regarded as the primary object. But many people appear to think that it could be as well fulfilled by the poor-law infirmary. To enter a poor-law infirmary, however, one must be a pauper, and as that name carries, and rightly carries, a certain social stigma with it, it is not fair to affix it to many who, for one reason or another, require medical or surgical treatment which cannot conveniently be given within their own homes. All accidents come under this heading. If a man, be he duke or dustman, is run over in the street, it is ten to one that he is conveyed to the nearest hospital; and, whatever his rank, he will be thankful that a place where his injuries can be promptly and skilfully treated is so near at hand. To convey him to his own home would involve a prolongation of acute pain, and the delay, the increased nervous exhaustion, and in many cases the less skilful dressing and nursing might cause an injury which, in itself, was not deadly, to take a serious or even a fatal turn. That many people died in former days, and die still in remote districts and uncivilised countries, from accidents which would be readily cured at a hospital, is too well known to need demonstration. We may, therefore, accept hospitals as a social necessity in cases of accident.

In many operation cases, also, it is difficult to see how hospitals could be dispensed with. Even the well to do, who can afford to pay for the best of medical and nursing skill, often prefer to have operations performed in hospitals. Illness makes demands that cannot be met in any but the most luxurious homes. That those who can afford it should desire to go to pay hospitals is only just; but we have to consider the case of the many who can, and do, meet all the ordinary expenses of life, but cannot bear the cost of specialists' fees and the constant attendance of specially-trained nurses. Not only the poor, but people in moderate circumstances are unable to bear these charges.

From the point of view of the public, who support the hospitals, what shall we say? Well, this is intimately connected with our other alternative, the allowing of the patient to die. Wherever, through hospital treatment, a man or a woman lives who but for it had died, we have the justification of the hospital. Wherever a man or a woman can work again, who but for hospital treatment would have been a burden on the charity of workers, we have a justification of hospitals tenfold stronger than the former one. If we are content that children should grow up as cripples, let us abolish children's hospitals. If we are content that whole families should fall into pauperism, because the father, the worker who helped the progress of the world in providing for the needs of his own family, is dead—dead of some not incurable disease, then let us abolish the hospitals where he might have been restored to health. If we are content that mothers should die and homes be broken up, because we grudge the cost of hospitals where the diseases to which their sex lays them open may be relieved, then let us abolish hospitals for women. But not unless we are content to increase by that amount the suffering of this world, the preventable deaths, the loss to society of workers, potential and actual, can we yet do without hospitals.

Of the other mission of hospitals there is not much to say. To cure disease we must study it. Who would willingly employ a doctor who had never seen a case of illness? We demand, and rightly, that those who treat us when we are ill should have seen similar cases and been taught how to deal with them under experienced teachers. And where can this instruction be given but in hospitals? The dispensary work which our medical students do is also useful, as showing them the conditions under which many of them must work in after life; but they cannot see there the best results, those which they must strive after. The hospital ward is the ideal sick room, which the student ever after carries in his mind's eye, and to which he tries to make the room of every patient conform. To see how often even the worst cases recover in these conditions is the best encouragement for him in his after career, and is the only way of training him, not only in the treatment of disease, but in the knowledge of these details of nursing and environment on which the success of treatment so often depends. And what is true of doctors is equally true of nurses. How are they to learn their work, except in a hospital, where they are carefully drilled in those apparent trifles on which success, which in this case means the health of the patient, may depend? Can we conceive of our elaborate arrangements for conquering disease having come to such perfection as they have attained, not to speak of those further developments we yet hope for, except by means of hospitals? Can anyone who has profited, directly or indirectly, by the knowledge of disease and its cure, that hospitals afford—and that includes everyone who has ever been sick—say that hospitals are less than a social necessity of our civilisation?

### SMALL SUBSCRIBERS TO HOSPITALS.

METROPOLITAN hospitals have always suffered, compared with provincial institutions, from the small sums received from annual subscriptions. Heretofore the metropolitan hospitals have depended too much upon one class of the community for their support. What is wanted is that each hospital should modify its method of appeal, so as to reach and secure annual subscribers of from 1s. to 10s. per annum. The Prince of Wales's Fund was established not only to start this system for the benefit of that fund, but to encourage individual hospitals to follow its example. A subscription book and stamp album, containing amongst many other things an autograph letter from the Princess of Wales, portraits of the Queen and the Prince of Wales, has been issued with the object of enabling subscribers of small sums of from 1s. to 10s. per annum to possess evidence of the fact that they are regular subscribers to the metropolitan hospitals. This subscription book and stamp album gives to every person who frequents the hospitals an opportunity of subscribing according to their means a small sum each year to these institutions.

Take an example. A hospital at the present time sends to Messrs. Simpkin, Marshall, and Co., 4, Stationers' Hall Court, E.C., the publishers of the stamps and subscription books, for a supply of these articles, which are invoiced to them accordingly, the stamps on sale or return, and the books at so much per dozen, the published price of each book being 6d. The money for all the stamps sold by the hospital will be credited to its funds, and at the end of the year a certificate signed by the treasurer will be sent to Messrs. Simpkin, Marshall, and Co., showing how many of the stamps received have been sold, and how many remain on hand. The Prince of Wales's Fund will then credit the hospital with the amount of stamps they have sold to subscribers, the proceeds of which were retained by the hospitals as and when received. The subscription books will be paid for either on delivery or at such time as may be mutually arranged between the publishers and each hospital. In the case of those hospitals where payments are taken from patients, this system offers great facilities for securing an army of small subscribers, seeing that the possession of the stamp album will keep the hospital constantly in mind, and be sure to induce the more careful of the patients to make an effort to give something regularly each year to the hospitals whether they are ill or not. In this way the revenue from annual subscribers to the London hospitals could easily be increased by £50,000.

### "The Daily Round."

To the sufferer the hospital is a place of rest—rest always from labour, rest often from pain, rest from anxiety as to the daily wants. Far otherwise is it with those by whose aid a hospital is managed. To them the hospital is a place of work, work which is steady, continuous, and exacting even in ordinary times, but in times of epidemics, or when great accidents occur, is overwhelming and trying to the strongest.

Let us consider some of the little incidents in the ordinary daily round of hospital work, things that are happening constantly, and see how they all work together for the good of the patients.

### Up to the Wards.

Here we have a frequent scene at every large hospital. On the stretcher in the lobby near the front entrance lies the victim of a street accident. "First aid" has been speedily rendered, and the patient is ready for removal to the ward. On arrival, he will be allowed to remain perfectly quiet for a time, and, when so far recovered from the shock as to allow of it, he will be tenderly nursed back to health and strength.

The porters whose duty it is to carry stretcher cases are steady and "seasoned" men. Many of them are ex-soldiers who have had years of experience and special training in carrying the sick, and they are accustomed to that discipline which makes them always ready to respond at the call of duty.

### Croup.

Croup! Yes; and what a terrible thing that is when it seizes upon the pet child! Yesterday well and running about; to-day hoarse and blue, and struggling for breath; to-morrow perhaps dead, stifled by the horrid membrane that fills up its windpipe. Croup, of all cases, requires quick and skilful treatment. Everything must be at hand—the doctors with the most modern instruments, the nurses, the steam kettles, the tent bed. But, besides what is actually done for the little patient, other things must be ready, so that at any moment, should the necessity arise, an operation may be done and the windpipe may be opened, and the air let in, and the little sufferer be saved from suffocation. For this, again, the most skilful help is necessary, and it is in the hospitals that this can be obtained. There are many moments in a doctor's life when he feels his power, and enjoys the satisfaction of mastering disease, but there is nothing—nothing in his whole life to come up to the gratification of doing a successful tracheotomy. A few minutes of careful operative work, a moment of sharp, nay, of terrible, anxiety, and then, all at once, the child—who up to that moment had been dying a most miserable death from suffocation, and had been spending its little weary remains of strength fighting and struggling for breath—suddenly drops into a peaceful sleep. One would think it was dead but for the returning colour and the gentle rise and fall of its little chest. And so it sleeps back into health. A house surgeon may well feel pride and contentment in his art when he pulls aside the curtains of the tent bed and sees his little patient sleeping peacefully. But it is part of the daily round, and it is for such triumphs over disease that the hospitals exist.

### Painful, but "Doesn't Mind."

One of the most curious things one meets with in the daily round is the self-control of the children and their power of bearing pain without flinching when once they feel confidence in those around them.

When the house surgeon touches this tender spot the child winces—who could help doing so?—but it "doesn't mind," for it knows its friends. Few people understand how much children will bear if they do but have faith, and nothing is more touching than the confidence which some little ones display in their "dressers." Those who think that medical students are but gay dogs, up to any sort of mischief, should go into the children's wards of our great hospitals. There they would see these medical students in quite a new light, laying themselves out in every possible way to lighten the loneliness of the little ones.

### A Bandaging Class.

Part of the daily round in all our great hospitals is the training of the nurses. Here we see half a dozen probationers receiving a lesson in the art of bandaging from a sister. The sister, as the reader will readily see, wears a dark serge dress, and is at the moment engaged in instructing one of the class in the intricacies of the ankle bandage. The figure on the table or bench is not a human being, it is merely a dummy kept for the sole purpose of being bandaged and bandaged and bandaged. Were "it" a fellow-creature what endless and peculiarly unique sufferings would "it" have to undergo. While Sister explains the ankle bandage to one, two of the others are practising on the dummy's head, and two others have prevailed upon an obviously good-natured colleague to submit to their more or less (nearly always less) tender handling. It is by no means so easy as it looks.

### The Dispensary Window.

Perhaps in no place can so many snatches of curious and mystifying conversation be heard as at the dispensary window of the out-patient department of a big hospital. All sorts and conditions gather there after being in the consulting-room of the medical man for advice as to their various ailments, and after receiving their prescriptions. They whisper audibly all manner of little oddities regarding members of their own or their neighbours' household, and they become as friendly and confidential in half an hour as if they had attended a funeral together in the same coach.

To many a patient the visit to the hospital is a great event, a landmark in their lives, for they often come long distances, and they come, not merely for their bottle of physic, but for "opinions," decisions, often whether they are to live or die. To those engaged in hospital work, however, these things are but part of the daily round. Suffering has to be relieved, disease has to be cured, advice has to be given, sympathetically and tenderly tales of misery and distress have to be listened to, and everything that can be done, to make the life of the sick easier and less weary, has to be done—just as part of the daily round. Surely the daily round of our great hospitals is a continual round of well-doing, and is worthy of every sympathy and every help.

### THE DISEASES FROM WHICH LONDONERS SUFFER.

Total, 1,755,042.

IN order to show the proportion borne to one another by the various classes of disease from which the inhabitants of London suffer, we have again this year prepared a set of diagrams, drawn to scale, showing the relative number of patients who applied to the hospitals in 1896 for the treatment of the different kinds of diseases set out under each square. The cases which we have sorted out in this way comprise those treated at all the voluntary hospitals and dispensaries of London—including the endowed hospitals, St. Bartholomew's, Guy's, and St. Thomas's—and also the hospitals of the Metropolitan Asylums Board. Altogether the patients amounted in 1896 to the enormous total of 1,755,042, represented by the large square which heads this page. Of these 662,084 were males above 15 years of age, 569,799 were females above 15, and 523,159 boys and girls aged from 15 years downwards. The area of all the other squares (excluding that for children, which has been inserted in order to show on the same scale the number of little ones treated at the special hospitals for children) put together equal the area of this large square. By far the largest is that which shows the relative size of the surgical cases treated at the hospitals, that is to say, of the cases treated for all kinds of injury which accident or pathological process may produce. As will be seen it is the equivalent of 828,276 patients, or nearly one-half of the whole number. Let anyone try to realise what this army of over 825,000 persons is, and they will see that one of the greatest benefits which the hospitals give to the sick poor of London lies in the facilities which they offer for the performance of surgical cures of a character undreamt of years ago. Next in size is the square relating to medical diseases, which represents 533,726 cases. That is to say that in 1896 considerably more than half a million human beings received medical treatment free of cost, at the hands of the leading physicians of the day, within the buildings of the hospitals of London.

Surgical Patients, 828,276.

Medical Patients, 533,726.

But this is not all. Besides the medical and surgical cases included in these two squares we have to reckon with the patients treated for diseases of the eye, for diseases peculiar to women and motherhood, for consumption, for affections of the nose, throat, and ear, for skin diseases, for fever, and for paralysis and diseases of the nervous system, for each of which there is an additional square, smaller by far it is true than the two diagrams with which we have been dealing, but each of them representing a very great amount of suffering. Take the first, that immediately below the medical patients. This, though apparently small, shows that there were 123,840 persons treated in the hospitals of London in 1896 for various forms of disease which often entailed excruciating pain, and must in many cases have terminated in loss of sight had it not been for the treatment they received at the hospitals. Then glance down to the next square on the left, that entitled "women." Small though it is, it represents no less than 81,000 women who have been attended to for the relief of diseases peculiar to their sex. Immediately underneath is shown the fact that 46,000 persons suffering from consumption, that curse of our climate, were treated in the London hospitals in 1896. Who can find it in his heart to deny something towards the cost of alleviating the sufferings of those who have been stricken with this terrible malady? The smallest square of all even is not one to be lightly passed over. It shows that nearly 16,000 people received treatment for epilepsy, paralysis, and diseases of the nerves. No disease is more appalling in its suddenness than paralysis, and it should be matter for thankfulness to Londoners that the hospitals and dispensaries of London were able during the year to give succour to these 16,000 cases. No one can doubt the association of nervous breakdown with the toil and moil of London life. Let those who are still spared give freely to relieve those who have fallen out of the race, and to keep open the hospitals where immediate treatment can be given to whomsoever may be struck down by those diseases. Space forbids our calling attention to all the other diagrams in detail, but the lesson they teach is, we think, sufficiently plain. If each person who reads this page will only try to realise what an amount of suffering relieved is represented by each of these little squares, we are sure that a liberal offering on Hospital Sunday will be the outcome of their endeavour.

Children, 124,039.

Eye, 123,848.

Women, 81,330.

Throat, 53,333.

Consumption, 46,120.

Skin, 43,823.

Fever, 28,802.

Paralysis, 15,784.

### Hospital Sunday in London, 12th June, 1898.

### THE WORK DONE BY THE HOSPITALS AND MEDICAL CHARITIES IN 1897.

### NEWINGTON AND SOUTH DISTRICT.

Comprising Battersea, Wandsworth, Tooting, Balham, Streatham, Brixton, Lambeth, Newington, Southwark, Bermondsey, Camberwell, Greenwich, Deptford, Lewisham, Blackheath, Woolwich, &c.

| No. of Beds. | No. of Beds Daily Occupied. | Hospitals. | In-patnts. | Out-patients. | Total Expenditure. | Income. | | | Total Income. | Legacies not included in previous column. |
|---|---|---|---|---|---|---|---|---|---|---|
| | | | | | | Charitable. | Proprietary. | Patnts' Pymnts | | |
| | | | | | £ | £ | £ | £ | £ | |
| 650 | 426 | Guy's ... ... ... ... ... ... ... | 6,177 | 80,098 | 46,930 | 15,453 | 29,956 | 4,487 | 49,896 | ... |
| 25 | 21 | Miller ... ... ... ... ... ... ... | 278 | 17,170 | 3,506 | 2,700 | 418 | ... | 3,118 | 190 |
| 253 | 203 | Seamen's... ... ... ... ... ... ... | 2,620 | 22,581 | 17,768 | 10,628 | 3,939 | 401 | 14,968 | 6,286 |
| 569 | 407 | St. Thomas's ... ... ... ... ... .. | 6 035 | 69,461 | 53,328 | 9,903 | 53,789 | 347 | 64,039 | 3,283 |
| 66 | 55 | Evelina, for Children ... ... ... ... | 862 | 8,944 | 5,390 | 5,505 | 704 | 72 | 6,281 | 500 |
| 57 | 51 | Home for Sick Children ... ... .. | 236 | 1,622 | 1,894 | 1,133 | 234 | 365 | 1,732 | 500 |
| 24 | 20 | General Lying-in ... ... ... ... | 520 | 1,845 | 3,364 | 767 | 2,609 | ... | 3,376 | ... |
| 36 | 14 | Clapham Maternity and Dispensary... ... | 337 | 5,075 | 2,078 | 500 | 965 | 806 | 2,271 | ... |
| 52 | 34 | Royal, for Children and Women ... ... | 375 | 6,525 | 3,773 | 3,260 | 805 | 176 | 4,241 | 500 |
| 42 | 19 | Royal Eye ... ... ... ... ... ... | 405 | 15,443 | 2,824 | 2,206 | 87 | 497 | 2,790 | 709 |
| 396 | 224 | Metropolitan Convalescent ... ... ... | 3,354 | ... | 7,571 | 4,624 | 344 | 239 | 5,207 | 1,300 |
| 10 | 5 | Phillips' Memorial Homœopathic ... ... | 74 | 2,910 | 549 | 346 | ... | 213 | 559 | ... |
| 8 | 4 | Eltham Cottage... ... ... ... ... | 57 | ... | 353 | 267 | 11 | 58 | 336 | ... |
| 20 | 14 | Beckenham Cottage ... ... ... ... | 159 | ... | 709 | 625 | 28 | 127 | 780 | ... |
| 18 | 11 | Blackheath Cottage ... ... ... ... | 160 | 1,113 | 836 | 696 | 20 | 95 | 811 | ... |
| 32 | 24 | Bromley Cottage ... ... ... ... ... | 300 | ... | 1,257 | 948 | 145 | 194 | 1,287 | 100 |
| 14 | 10 | Chislehurst, &c., Cottage ... ... ... | 147 | ... | 606 | 573 | 31 | 148 | 752 | ... |
| 12 | 7 | Sidcup Cottage... ... ... ... ... | 106 | 44 | 502 | 342 | ... | 93 | 435 | ... |
| 7 | 6 | Shortlands Convalescent ... ... ... | 108 | ... | 175 | 171 | 2 | 2 | 175 | ... |
| 10 | 6 | Woolwich and Plumstead Cottage ... .. | 73 | ... | 459 | 364 | 10 | 78 | 452 | ... |
| 2,301 | 1,561 | | 22,383 | 232,831 | 153,872 | 61,011 | 94,097 | 8,398 | 163,506 | 13,368 |
| | | DISPENSARIES. | | | | | | | | |
| ... | ... | Battersea Provident ... ... ... ... | ... | 25,575 | 3,165 | 85 | 60 | 2,950 | 3,095 | ... |
| ... | ... | Brixton, &c. ... ... ... ... ... | ... | 4,137 | 635 | 583 | 18 | 90 | 691 | ... |
| ... | ... | Camberwell Provident ... ... ... | ... | 11,196 | 1,939 | 374 | 131 | 1,507 | 2,012 | ... |
| ... | ... | Clapham ... ... ... ... ... ... | ... | 1,502 | 393 | 218 | ... | 110 | 328 | ... |
| ... | ... | Deptford Medical Mission ... ... ... | ... | 2,710 | 435 | 303 | 43 | 82 | 425 | ... |
| ... | ... | East Dulwich Provident ... ... ... | ... | 5,268 | 701 | 79 | 1 | 634 | 714 | ... |
| ... | ... | Forest Hill ... ... ... ... ... | ... | 2,398 | 713 | 215 | 4 | 465 | 684 | ... |
| ... | ... | Royal South London ... ... ... ... | ... | 4,695 | 762 | 530 | 112 | 5 | 647 | ... |
| ... | ... | South Lambeth, &c. ... ... ... ... | ... | 2,116 | 556 | 329 | 15 | 170 | 514 | ... |
| ... | ... | Walworth Provident ... ... ... ... | ... | 656 | 237 | 13 | 30 | 130 | 170 | ... |
| ... | ... | Wandsworth Common... ... ... ... | ... | 888 | 194 | 47 | ... | 147 | 194 | ... |
| ... | ... | Woolwich, &c., Provident ... ... ... | ... | 751 | 253 | 29 | 13 | 145 | 187 | ... |
| 2,301 | 1,561 | | 22,383 | 294,723 | 163,855 | 63,810 | 94,524 | 14,833 | 173,167 | 13,368 |

### CITY AND EAST CENTRAL DISTRICT.

Comprising the City, St. Luke's, Shoreditch, Finsbury, and Clerkenwell.

| No. of Beds. | No. of Beds Daily Occupied. | Hospitals. | In-patints. | Out-patients. | Total Expenditure. | Income. | | | Total Income. | Legacies not included in previous column. |
|---|---|---|---|---|---|---|---|---|---|---|
| | | | | | | Charitable. | Proprietary. | Patnts' pymnts. | | |
| | | | | | £ | £ | £ | £ | £ | £ |
| 114 | 70 | Metropolitan ... ... ... ... ... | 1,055 | 27,807 | 9,367 | 6,215 | 619 | 198 | 7,032 | 1,113 |
| 170 | 143 | Royal Free ... ... ... ... ... | 2,020 | 33,346 | 12,149 | 5,259 | 1,214 | ... | 6,473 | 3,664 |
| 80 | 58 | Royal, for Diseases of the Chest ... ... | 674 | 6,356 | 7,675 | 5,126 | 150 | ... | 5,276 | 900 |
| 57 | 49 | North-Eastern, for Children ... ... | 772 | 14,947 | 5,662 | 4,343 | 606 | 661 | 5,610 | ... |
| 36 | 23 | City of London Lying-In ... ... ... | 521 | 1,668 | 3,864 | 720 | 3,479 | 6 | 4,205 | 69 |
| 45 | 26 | St. Mark's, for Fistula ... ... ... | 293 | 886 | 3,223 | 1,251 | 639 | ... | 1,890 | 579 |
| 100 | 70 | Royal London Ophthalmic ... ... ... | 1,968 | 25,051 | 6,380 | 3,091 | 607 | ... | 3,698 | 1,374 |
| 45 | 34 | City Orthopædic ... ... ... ... | 202 | 2,274 | 1,478 | 1,691 | 33 | ... | 1,724 | ... |
| 17 | 9 | Central London Throat and Ear ... ... | 277 | 5,682 | 1,569 | 274 | 50 | 1,101 | 1,425 | ... |
| 664 | 482 | DISPENSARIES. | 7,782 | 118,017 | 51,637 | 27,970 | 7,397 | 1,966 | 37,333 | 7,999 |
| ... | ... | City ... ... ... ... ... ... | ... | 4,570 | 1,933 | 1,215 | 158 | ... | 1,373 | ... |
| ... | ... | City of London and East London ... ... | ... | 16,864 | 1,374 | 121 | 52 | 1,524 | 1,697 | ... |
| ... | ... | Farringdon General ... ... ... ... | ... | 4,329 | 637 | 203 | ... | 262 | 465 | ... |
| ... | ... | Finsbury... ... ... ... ... ... | ... | 15,421 | 949 | 438 | 145 | 299 | 882 | ... |
| ... | ... | Metropolitan ... ... ... ... ... | ... | 6,015 | 865 | 552 | 117 | 301 | 970 | ... |
| ... | ... | Royal General ... ... ... ... ... | ... | 2,949 | 827 | 318 | 345 | 93 | 756 | ... |
| 664 | 482 | | 7,782 | 168,165 | 58,222 | 30,817 | 8,214 | 4,445 | 43,476 | 7,999 |

### ST. MARYLEBONE AND WEST CENTRAL DISTRICT.

Comprising St. Marylebone, St. John's Wood, Bloomsbury, Holborn, &c.

| No. of Beds. | No. of Beds Daily Occupied. | HOSPITALS. | In-patnts. | Out-patients. | Total Expenditure. | Income. Charitable. | Income. Proprietary. | Income. Patnts' pymnts. | Total Income. | Legacies not included in previous column. |
|---|---|---|---|---|---|---|---|---|---|---|
| | | | | | £ | £ | £ | £ | £ | £ |
| 70 | 43 | French ... ... ... ... ... ... ... | 711 | 4,722 | 4,961 | 3,934 | 104 | ... | 4,038 | 2,261 |
| 20 | 6 | Italian ... ... ... ... ... ... ... | 122 | 4,617 | 839 | 1,632 | 93 | ... | 1,725 | ... |
| 100 | 77 | London Homœopathic ... ... ... ... | 1,064 | 16,899 | 12,602 | 3,272 | 3,096 | 687 | 7,055 | 1,217 |
| 51 | 34 | SS. John and Elizabeth ... ... ... | 51 | ... | 1,812 | 1,177 | 951 | ... | 2,128 | 2,393 |
| 321 | 270 | The Middlesex ... ... ... ... ... ... | 3,641 | 47,252 | 30,759 | 12,206 | 8,640 | ... | 20,846 | 19,250 |
| 68 | 57 | Alexandra for Children ... ... ... | 101 | 290 | 2,649 | 2,129 | 127 | 407 | 2,663 | ... |
| 30 | 26 | Hospital for Incurable Children ... ... | 35 | ... | 1,157 | 486 | 48 | 411 | 945 | 1,350 |
| 238 | 159 | Hospital for Sick Children ... ... ... | 2,145 | 23,673 | 15,937 | 7,056 | 3,288 | 24 | 10,368 | 7,057 |
| 16 | 9 | British Lying-In ... ... ... ... ... | 187 | 390 | 1,578 | 532 | 1,269 | 28 | 1,829 | ... |
| 68 | 49 | Queen Charlotte's Lying-In ... ... ... | 1,194 | 1,124 | 4,475 | 3,101 | 1,196 | 135 | 4,432 | 1,100 |
| 42 | 38 | New Hospital for Women ... ... ... | 555 | 6,831 | 4,739 | 2,292 | 176 | 1,474 | 3,942 | 1,050 |
| 50 | 48 | Samaritan Free... ... ... ... ... | 542 | 8,433 | 6,747 | 5,961 | 149 | ... | 6,110 | 920 |
| 200 | 163 | National for the Paralysed, &c. ... ... | 1,001 | 5,939 | 14,260 | 5,133 | 1,691 | 1,899 | 8,723 | 9,677 |
| 25 | 19 | Hospital for Epilepsy, &c. ... ... ... | 81 | 765 | 1,933 | 950 | 50 | 623 | 1,623 | 94 |
| 50 | 36 | West End, for Epilepsy, &c.... ... ... | 296 | 3,021 | 2,864 | 1,996 | 69 | 482 | 2,547 | ... |
| 13 | 9 | Central London Ophthalmic ... ... ... | 209 | 10,579 | 1,347 | 823 | 8 | 458 | 1,289 | ... |
| 15 | 5 | Western Ophthalmic ... ... ... ... | 126 | 7,590 | 1,053 | 443 | 154 | ... | 597 | ... |
| 60 | 50 | National Orthopædic ... ... ... ... | 207 | 1,229 | 2,269 | 974 | ... | 1,015 | 1,989 | 150 |
| 25 | 15 | Establishment for Gentlewomen ... ... | 131 | ... | 2,280 | 885 | 118 | 890 | 1,893 | 500 |
| ... | ... | National Dental ... ... ... ... | ... | 29,454 | 1,229 | 1,339 | ... | 166 | 1,505 | ... |
| 14 | 4 | London Throat ... ... ... ... ... | 298 | 4,191 | 1,170 | 419 | ... | 779 | 1,198 | ... |
| 1,471 | 1,117 | DISPENSARIES. | 12,697 | 176,999 | 116,660 | 56,840 | 21,207 | 9,498 | 87,545 | 47,019 |
| ... | ... | Bloomsbury Provident ... ... ... ... | ... | 897 | 257 | 22 | ... | 191 | 213 | ... |
| ... | ... | London Medical Mission ... ... ... ... | ... | 5,080 | 1,233 | 799 | 82 | 205 | 1,086 | 50 |
| ... | ... | Portland Town... ... ... ... ... ... | ... | 1,750 | 181 | 101 | 5 | 12 | 118 | ... |
| ... | ... | Infirmary for Consumption ... ... ... ... | ... | 1,080 | 382 | 257 | 187 | ... | 444 | ... |
| ... | ... | St. John's Wood Provident ... ... ... ... | ... | 6,227 | 628 | 255 | 32 | 367 | 654 | ... |
| ... | ... | St. Marylebone General ... ... ... ... | ... | 3,663 | 980 | 426 | 175 | 316 | 917 | ... |
| ... | ... | Western General ... ... ... ... ... | ... | 14,348 | 1,228 | 1,142 | 31 | 38 | 1,211 | ... |
| 1,471 | 1,117 | | 12,697 | 210,044 | 121,541 | 59,842 | 21,719 | 10,627 | 92,188 | 47,069 |

### KENSINGTON AND WEST DISTRICT.

Comprising Kensington, Paddington, Bayswater, Kilburn, Chelsea, Brompton, Fulham, Hammersmith, Chiswick, Brentford, Acton, Ealing, &c.

| No. of Beds. | No. of Beds Daily Occupied. | HOSPITALS. | In-patnts. | Out-patients. | Total Expenditure. | Income. Charitable. | Income. Proprietary. | Income. Patnts' pymnts. | Total Income. | Legacies not included in previous column. |
|---|---|---|---|---|---|---|---|---|---|---|
| | | | | | £ | £ | £ | £ | £ | £ |
| 351 | 327 | St. George's ... ... ... ... ... ... ... | 4,249 | 26,481 | 44,222 | 15,827 | 15,597 | ... | 31,424 | 19,301 |
| 281 | 200 | St. Mary's ... ... ... ... ... ... ... | 3,317 | 36,425 | 21,816 | 12,921 | 2,134 | ... | 15,055 | 17,870 |
| 97 | 90 | West London ... ... ... ... ... ... | 1,591 | 35,241 | 7,250 | 6,377 | 253 | ... | 6,630 | 1,130 |
| 321 | 238 | Hospital for Consumption ... ... ... ... | 1,408 | 7,630 | 36,216 | 13,532 | 7,907 | ... | 21,439 | 5,970 |
| 24 | 20 | Belgrave, for Children ... ... ... ... | 327 | 4,743 | 1,521 | 1,310 | 97 | ... | 1,407 | 200 |
| 50 | 50 | Cheyne, for Sick & Incurable Children ... | 73 | ... | 2,200 | 1,731 | 212 | 456 | 2,399 | ... |
| 46 | 33 | Paddington Green, for Children ... ... | 537 | 14,296 | 3,408 | 2,637 | 148 | 262 | 3,047 | 50 |
| 120 | 93 | Victoria, for Children... ... ... ... | 1,406 | 17,719 | 8,645 | 5,703 | 483 | 408 | 6,594 | 190 |
| 52 | 45 | Chelsea, for Women ... ... ... ... | 661 | 2,619 | 4,410 | 3,116 | 232 | 786 | 4,134 | ... |
| 105 | 83 | Cancer ... ... ... ... ... ... ... | 832 | 1,589 | 11,840 | 5,387 | 2,584 | ... | 7,971 | 9,736 |
| 135 | 88 | Female Lock ... ... ... ... ... ... | 694 | ... | 4,118 | 2,163 | ... | 1,335 | 3,498 | 445 |
| 16 | 8 | Epsom and Ewell Cottage ... ... ... | 97 | ... | 837 | 551 | 8 | 173 | 732 | ... |
| 20 | 17 | Reigate and Redhill Cottage... ... ... | 222 | ... | 846 | 635 | 53 | 133 | 821 | 10 |
| 11 | 6 | Wimbledon Cottage ... ... ... ... | 103 | ... | 489 | 505 | 5 | 72 | 582 | ... |
| 14 | 9 | Hounslow Cottage ... ... ... ... ... | 79 | 648 | 400 | 284 | 174 | 45 | 503 | 100 |
| 1,643 | 1,307 | DISPENSARIES. | 15,596 | 147,391 | 148,218 | 72,679 | 29,887 | 3,670 | 106,236 | 55,002 |
| ... | ... | Brompton Provident ... ... ... ... ... | ... | 1,691 | 462 | 134 | 81 | 242 | 457 | ... |
| ... | ... | Chelsea, &c. ... ... ... ... ... ... | ... | 4,518 | 672 | 507 | 181 | ... | 688 | ... |
| ... | ... | Chelsea Provident ... ... ... ... ... | ... | 531 | 264 | 15 | 13 | 157 | 185 | ... |
| ... | ... | Kensal Town Provident ... ... ... ... | ... | 784 | 321 | 21 | 48 | 236 | 305 | ... |
| ... | ... | Kensington ... ... ... ... ... ... | ... | 3,796 | 629 | 604 | 49 | ... | 653 | 50 |
| ... | ... | Kilburn, Maida Vale ... ... ... ... | ... | 1,702 | 560 | 370 | 51 | ... | 421 | ... |
| ... | ... | Kilburn Provident ... ... ... ... ... | ... | 4,303 | 1,107 | 63 | 6 | 1,037 | 1,106 | ... |
| ... | ... | Notting Hill Provident ... ... ... ... | ... | 256 | 183 | 69 | ... | 61 | 130 | ... |
| ... | ... | Paddington Provident ... ... ... ... | ... | 3,500 | 551 | 135 | 26 | 369 | 530 | ... |
| ... | ... | Pimlico Provident ... ... ... ... ... | ... | 2,350 | 721 | 14 | ... | 708 | 722 | ... |
| ... | ... | Royal Pimlico Provident ... ... ... ... | ... | 6,877 | 1,065 | 345 | 38 | 534 | 917 | ... |
| ... | ... | Westbourne Provident ... ... ... ... | ... | 980 | 400 | 47 | 43 | 325 | 415 | ... |
| 1,643 | 1,307 | | 15,596 | 178,679 | 155,153 | 75,003 | 30,423 | 7,339 | 112,765 | 55,052 |

### ISLINGTON AND NORTH-WEST DISTRICT.

Comprising Islingto^, Holloway, Highbury, Hampstead, Highgate, St. Pancras, Stoke Newington, Tottenham, &c.

| No. of Beds. | No. of Beds Daily Occupied. | Hospitals. | In-patnts. | Out-patients. | Total Expenditure. | Income. Charitable. | Income. Proprietary. | Income. Patnts' pymnts | Total Income. | Legacies not included in previous column. |
|---|---|---|---|---|---|---|---|---|---|---|
| | | | | | £ | £ | £ | £ | £ | £ |
| 107 | 101 | Great Northern Central ... ... ... ... | 1,527 | 23,795 | 10,257 | 7,501 | 833 | 556 | 8,890 | 713 |
| 30 | 20 | Hampstead Hospital ... ... ... ... | 305 | 740 | 2,873 | 2,590 | 668 | 374 | 3,632 | ... |
| 120 | 68 | London Temperance ... ... ... ... | 1,175 | 18,387 | 11,196 | 12,774 | 913 | 309 | 13,996 | 1,160 |
| 52 | 45 | North-West London ... ... ... ... | 560 | 17,276 | 4,064 | 3,360 | 175 | 48 | 3,583 | ... |
| 70 | 38 | Tottenham Training ... ... ... ... | 389 | 6,891 | 3,186 | 1,242 | 303 | 140 | 1,685 | ... |
| 206 | 160 | University College ... ... ... ... | 2,875 | 50,097 | 18,053 | 13,908 | 3,279 | 52 | 17,239 | 2,663 |
| 60 | 54 | North London Consumption ... ... ... | 430 | 3,279 | 5,254 | 5,014 | 116 | 28 | 5,158 | .. |
| 150 | 71 | London Fever ... ... ... ... ... | 579 | ... | 14,719 | 7,819 | 1,643 | 1,891 | 11,253 | 3,752 |
| 28 | 18 | Invalid Asylum ... ... ... ... ... | 220 | ... | 943 | 520 | 133 | 190 | 862 | ... |
| 16 | 14 | Children's Home Hospital, Barnet ... .. | 66 | ... | 585 | 497 | 35 | 90 | 622 | ... |
| 10 | 8 | Enfield Cottage ... ... ... ... .. | 108 | ... | 492 | 579 | 26 | 67 | 672 | ... |
| 30 | 25 | Memorial Cottage, Mildmay ... .. | 161 | ... | 1,678 | 686 | 941 | 176 | 1,803 | ... |
| 28 | 14 | St. Saviour's Home ... ... ... .. | 83 | ... | 1,757 | 1,085 | 4 | 560 | 1,649 | 20 |
| 40 | 28 | Friedenheim Home ... ... ... ... | 85 | ... | 3,423 | 3,082 | 77 | 251 | 3,410 | ... |
| 32 | 22 | St. Monica's, Brondesbury ... ... ... | 37 | ... | 1,539 | 1,425 | 195 | 286 | 1,906 | ... |
| 979 | 686 | Dispensaries. | 8,600 | 120,465 | 80,019 | 62,101 | 9,141 | 5,018 | 76,260 | 8,308 |
| ... | ... | Child's Hill Provident .. ... ... ... | ... | 466 | 327 | 35 | 18 | 275 | 338 | ... |
| ... | ... | Camden Provident ... ... ... ... | ... | 1,032 | 288 | 19 | ... | 232 | 251 | ... |
| ... | ... | Hampstead Provident ... ... ... ... | ... | 10,991 | 999 | 231 | 53 | 725 | 1,009 | ... |
| ... | ... | Holloway and North Islington ... ... | ... | 2,818 | 568 | 189 | 34 | 272 | 495 | ... |
| ... | ... | Islington ... ... ... ... ... .. | ... | 12,100 | 867 | 293 | 21 | 568 | 882 | ... |
| ... | ... | St. Pancras and Northern ... ... ... | ... | 1,804 | 566 | 267 | 108 | 98 | 473 | ... |
| ... | ... | Stamford Hill, &c. ... ... ... ... | ... | 3,948 | 626 | 493 | 163 | ... | 656 | 46 |
| 979 | 686 | | 8,600 | 153,624 | 84,260 | 63,628 | 9,538 | 7,188 | 80,354 | 8,354 |

### WESTMINSTER DISTRICT. Comprising Westminster City and Liberties.

| No. of Beds. | No. of Beds Daily Occupied. | Hospitals. | In-patnts. | Out-patients. | Total Expenditure. | Charitable. | Proprietary. | Patnts' pymnts | Total Income. | Legacies not included in previous column. |
|---|---|---|---|---|---|---|---|---|---|---|
| | | | | | £ | £ | £ | £ | £ | £ |
| 175 | 142 | Charing Cross ... ... ... ... ... | 2,017 | 23,651 | 16,138 | 32,276 | 1,301 | ... | 34,577 | 19,463 |
| 217 | 178 | King's College ... ... ... ... ... | 2,419 | 24,501 | 18,665 | 12,603 | 2,317 | 65 | 14,985 | 6,219 |
| 202 | 173 | Westminster ... ... ... ... ... | 2,620 | 23,101 | 16,545 | 6,166 | 2,969 | ... | 9,135 | 4,862 |
| 134 | 124 | Ventnor, for Consumption ... ... ... | 848 | ... | 13,422 | 7,545 | 1,886 | 3,655 | 13,080 | ... |
| 7 | 5 | Grosvenor, for Women and Children ... | 91 | 2,617 | 1,310 | 856 | 4 | 300 | 1,160 | ... |
| 61 | 49 | Hospital for Women ... ... ... ... | 720 | 4,952 | 6,669 | 3,156 | 341 | 447 | 3,944 | 10 |
| 25 | 20 | National, for Diseases of Heart, &c.... ... | 141 | 1,836 | 2,134 | 1,675 | 33 | 298 | 2,006 | 1,000 |
| 30 | 25 | Royal Westminster Ophthalmic ... ... | 623 | 10,367 | 2,262 | 2,113 | 463 | 85 | 2,661 | ... |
| 50 | 47 | Royal Orthopædic ... ... ... .. | 170 | 721 | 1,843 | 769 | 523 | 265 | 1,557 | ... |
| 9 | 8 | Royal Ear ... ... ... ... ... | 307 | 2,047 | 795 | 279 | ... | 612 | 891 | ... |
| ... | ... | Dental ... ... ... ... ... ... | ... | 38,530 | 2,104 | 2,245 | 235 | 197 | 2,677 | ... |
| 20 | 15 | Gordon, for Fistula ... ... ... .. | 253 | 923 | 1,364 | 489 | ... | 885 | 1,374 | ... |
| 27 | 19 | St. Peter's, for Stone ... ... ... ... | 430 | 4,858 | 3,568 | 743 | 322 | 2,172 | 3,237 | ... |
| 957 | 805 | Dispensaries. | 10,639 | 137,413 | 86,819 | 71,915 | 10,388 | 8,981 | 91,284 | 31,554 |
| ... | ... | Public ... ... ... ... ... ... | ... | 3,114 | 774 | 408 | 180 | ... | 588 | ... |
| ... | ... | St. George and St. James ... ... .. | ... | 4,238 | 513 | 441 | 13 | 23 | 477 | ... |
| ... | ... | St. George, Hanover Square ... ... ... | ... | 859 | 534 | 404 | ... | 126 | 530 | ... |
| ... | ... | Western ... ... ... ... ... ... | ... | 8,005 | 1,438 | 493 | 412 | 609 | 1,514 | ... |
| ... | ... | Westminster General ... ... ... ... | ... | 6,803 | 737 | 431 | 163 | 114 | 708 | 45 |
| 957 | 805 | | | 160,432 | 90,815 | 74,092 | 11,156 | 9,853 | 95,101 | 31,599 |

### STRATFORD AND EAST-END DISTRICT.

Comprising Bethnal Green, Tower Hamlets, West Ham, Whitechapel, Hackney, Stepney, Limehouse, Poplar, and the East.

| No. of Beds. | No. of Beds Daily Occupied. | Hospitals. | In-patnts. | Out-patients. | Total Expenditure. | Charitable. | Proprietary. | Patnts' pymnts | Total Income. | Legacies not included in previous column. |
|---|---|---|---|---|---|---|---|---|---|---|
| | | | | | £ | £ | £ | £ | £ | £ |
| 125 | 100 | German ... ... ... ... ... ... | 1,498 | 20,442 | 12,514 | 6,194 | 2,391 | 394 | 8,979 | 51 |
| 776 | 634 | London ... ... ... ... ... ... | 10,501 | 161,033 | 77,742 | 28,381 | 25,063 | 90 | 53,534 | 20,888 |
| 65 | 52 | Poplar ... ... ... ... ... ... | 1,037 | 19,045 | 11,755 | 6,200 | 850 | 114 | 7,164 | 1,000 |
| 60 | 40 | West Ham, &c. ... ... ... ... ... | 657 | 20,985 | 4,390 | 4,039 | 182 | ... | 4,221 | ... |
| 34 | 21 | Walthamstow, &c. ... ... ... ... | 354 | 350 | 1,616 | 1,721 | 441 | ... | 2,162 | ... |
| 164 | 80 | City of London for Diseases of the Chest .. | 616 | 15,829 | 9,225 | 6,684 | 225 | ... | 6,909 | 2,175 |
| 102 | 86 | East London for Children ... ... ... | 1,616 | 33,468 | 10,200 | 6,291 | 1,042 | ... | 7,333 | 5,494 |
| 92 | 35 | Mrs. Gladstone's Home ... ... ... | 647 | ... | 990 | 413 | 377 | ... | 790 | 369 |
| 13 | 9 | East End Mother's Home ... ... ... | 237 | 241 | 1,388 | 1,018 | 397 | 44 | 1,459 | ... |
| 50 | 34 | Mildmay Mission Hospital ... ... .. | 442 | 6,890 | 3,869 | 2,742 | 581 | 47 | 3,370 | ... |
| 30 | 26 | St. Mary's, Plaistow ... ... ... ... | 481 | 6,084 | 2,088 | 1,765 | 141 | 31 | 1,937 | ... |
| 1,511 | 1,117 | Dispensaries. | 18,086 | 284 367 | 134,977 | 65,448 | 31,690 | 720 | 97,858 | 29,977 |
| ... | ... | Eastern ... ... ... ... ... ... | ... | 5,919 | 639 | 410 | 332 | 72 | 814 | ... |
| ... | ... | Hackney Provident ... ... ... ... | ... | 812 | 256 | 48 | ... | 183 | 231 | ... |
| ... | ... | London ... ... ... ... ... ... | ... | 2,276 | 701 | 142 | 304 | ... | 446 | ... |
| ... | ... | Queen Adelaide's ... ... ... ... | ... | 6,725 | 513 | 310 | 213 | 34 | 557 | ... |
| ... | ... | Tower Hamlets ... ... ... ... ... | ... | 3,935 | 600 | 312 | 27 | 146 | 485 | ... |
| ... | ... | Whitechapel Provident ... ... ... | ... | 4,968 | 807 | 18 | ... | 756 | 774 | ... |
| 1,511 | 1,117 | | 18,086 | 309,002 | 138,493 | 66,688 | 32,566 | 1,911 | 101,165 | 29,977 |

### THE METROPOLITAN HOSPITALS.—A SUMMARY OF THE WORK DONE IN 1897.

It will be seen from the following summary that the Voluntary Hospitals and Medical Charities of London, during the twelve months ending 31st December, 1897, relieved over one million five hundred and seventy thousand patients, at a cost of £812,339. The Ordinary Income only amounted to £698,216, leaving a deficiency of £114,123 on the year's work. The Legacies received in 1897 amounted to £193,418, being £61,037 in excess of the amount received in 1896.

| No. of Beds. | No. of Beds Daily Occupied. | Hospitals and Dispensaries. | In-patients. | Out-patients. | Total Expenditure. | Income. | | | Total Income. | Legacies not included in previous column. |
|---|---|---|---|---|---|---|---|---|---|---|
| | | | | | | Charitable. | Proprietary. | Patients' Payments. | | |
| | | | | | £ | £ | £ | £ | £ | £ |
| 2,301 | 1,561 | Newington and South District .. | 22,383 | 294,723 | 163,855 | 63,810 | 94,524 | 14,833 | 173,167 | 13,368 |
| 664 | 482 | City and East Central District .. | 7,782 | 168,165 | 58,222 | 30,817 | 8,214 | 4,445 | 43,476 | 7,999 |
| 57 | 805 | Westminster District ... ... | 10,639 | 160,432 | 90,815 | 74,092 | 11,156 | 9,853 | 95,101 | 31,599 |
| 1,471 | 1,117 | St. Marylebone and West Central District ... ... ... ... | 12,697 | 210,044 | 121,541 | 59,842 | 21,719 | 10,627 | 92,188 | 47,069 |
| 1,643 | 1,307 | Kensington and West District ... | 15,596 | 178,679 | 155,153 | 75,003 | 30,423 | 7,339 | 112,765 | 55,052 |
| 979 | 681 | Islington and North-West District | 8,600 | 150,624 | 84,260 | 63,628 | 9,538 | 7,188 | 80,354 | 8,354 |
| 1,511 | 1,117 | Stratford and East-end District ... | 18,086 | 309,002 | 138,493 | 66,688 | 32,566 | 1,911 | 101,165 | 29,977 |
| 9,526 | 7,075 | | 95,783 | 1,474,669 | 812,339 | 433,880 | 208,140 | 56,196 | 698,216 | 193,418 |

## Figures and Tables

**Figure f1:**
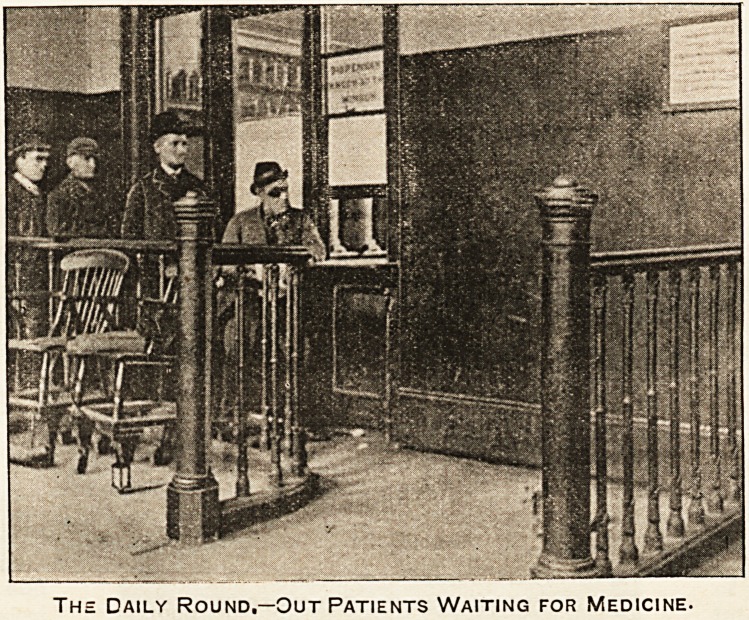


**Figure f2:**
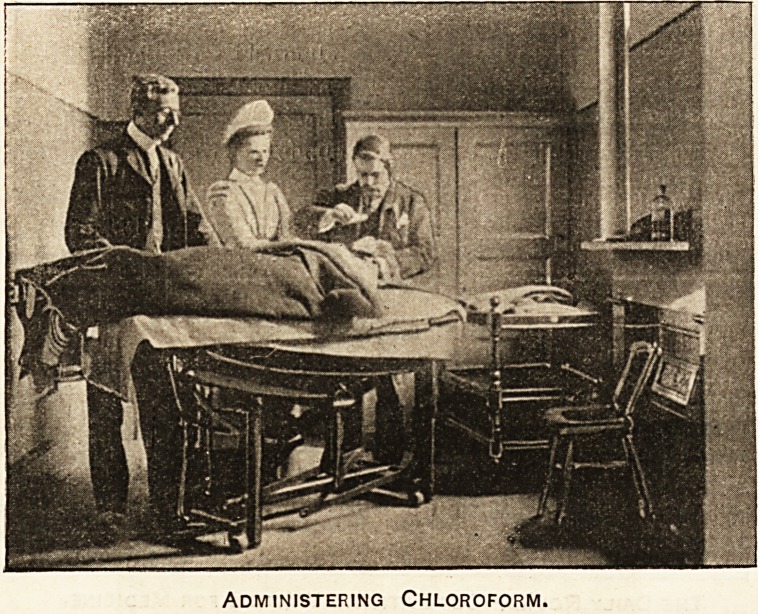


**Figure f3:**
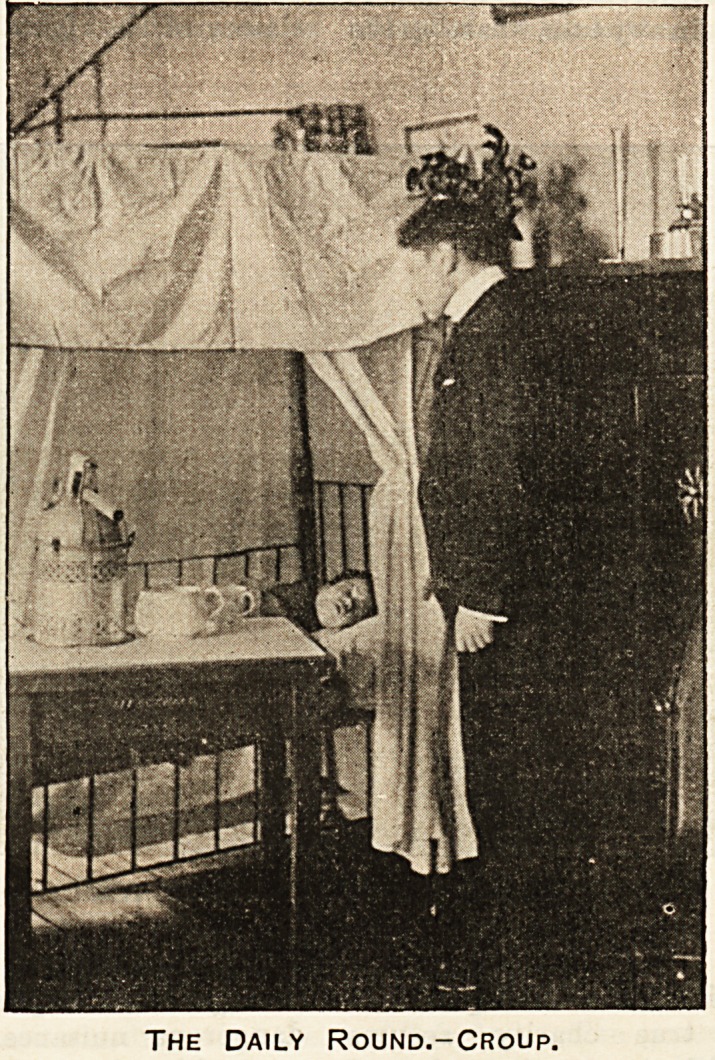


**Figure f4:**
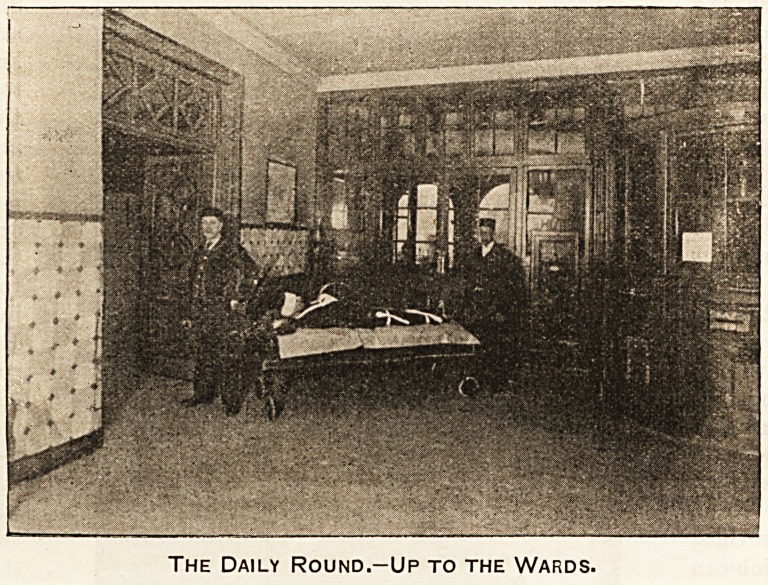


**Figure f5:**
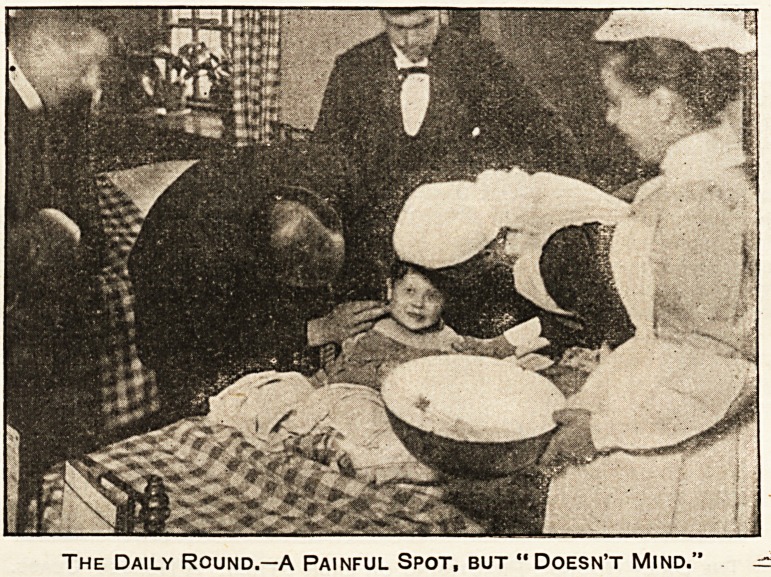


**Figure f6:**
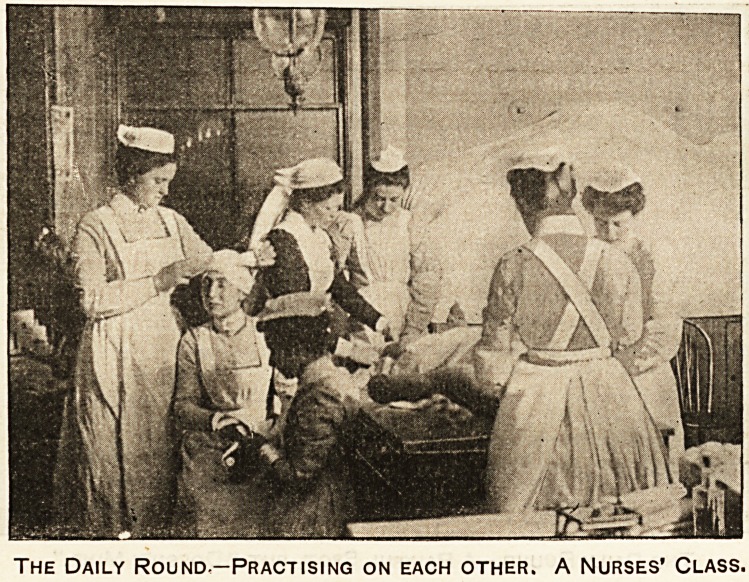


**Figure f7:**
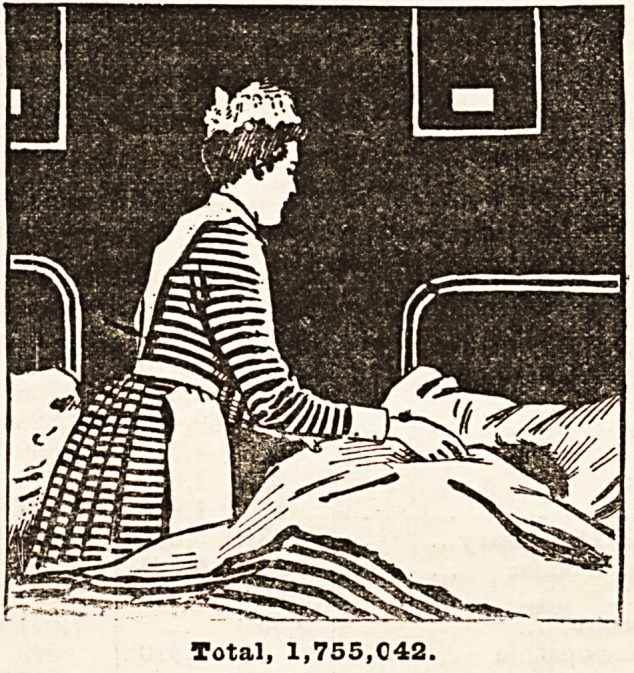


**Figure f8:**
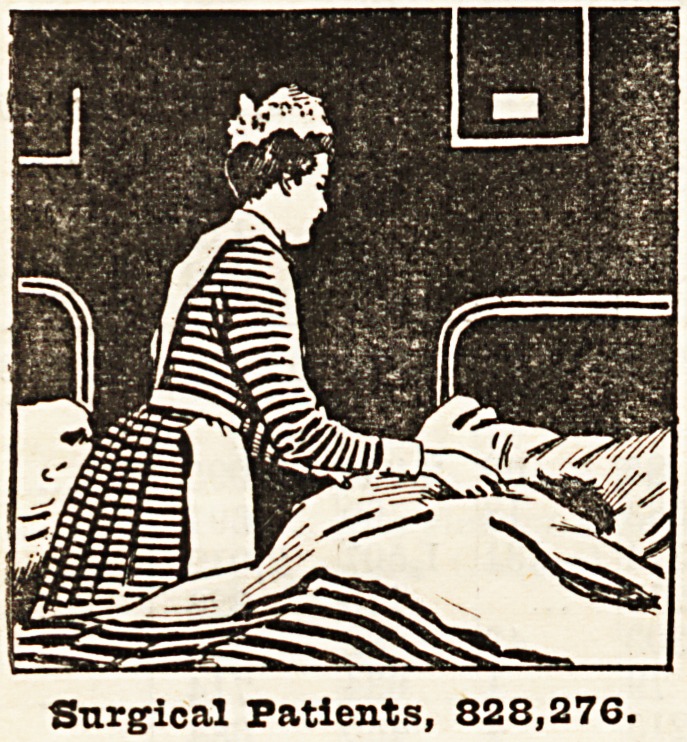


**Figure f9:**
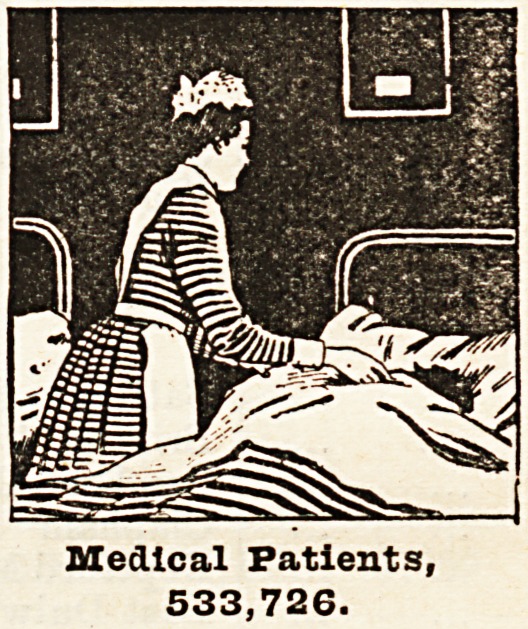


**Figure f10:**
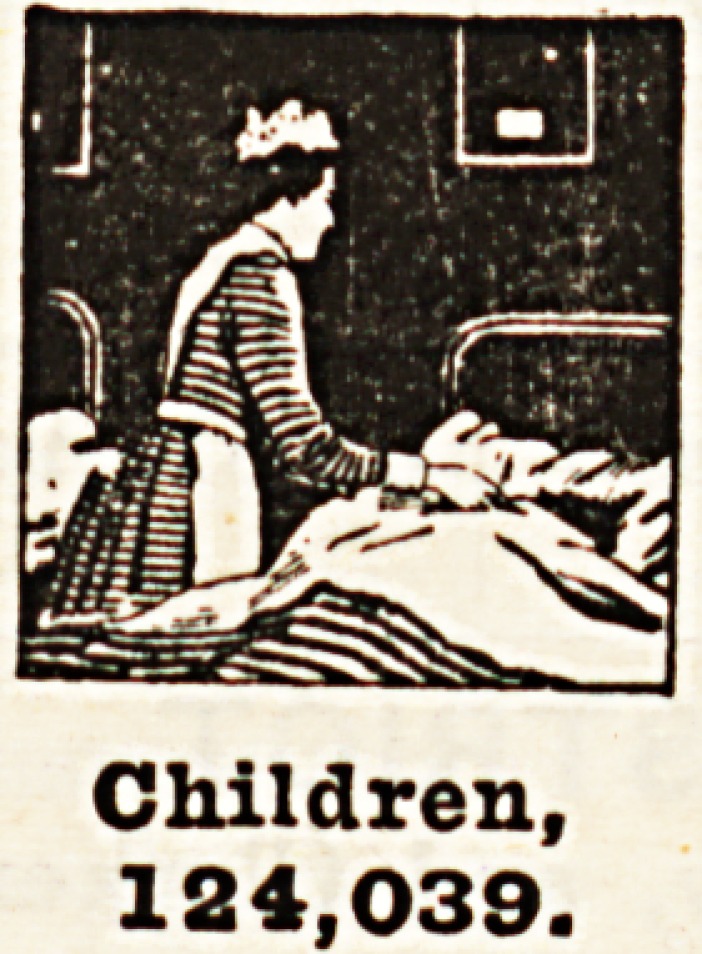


**Figure f11:**
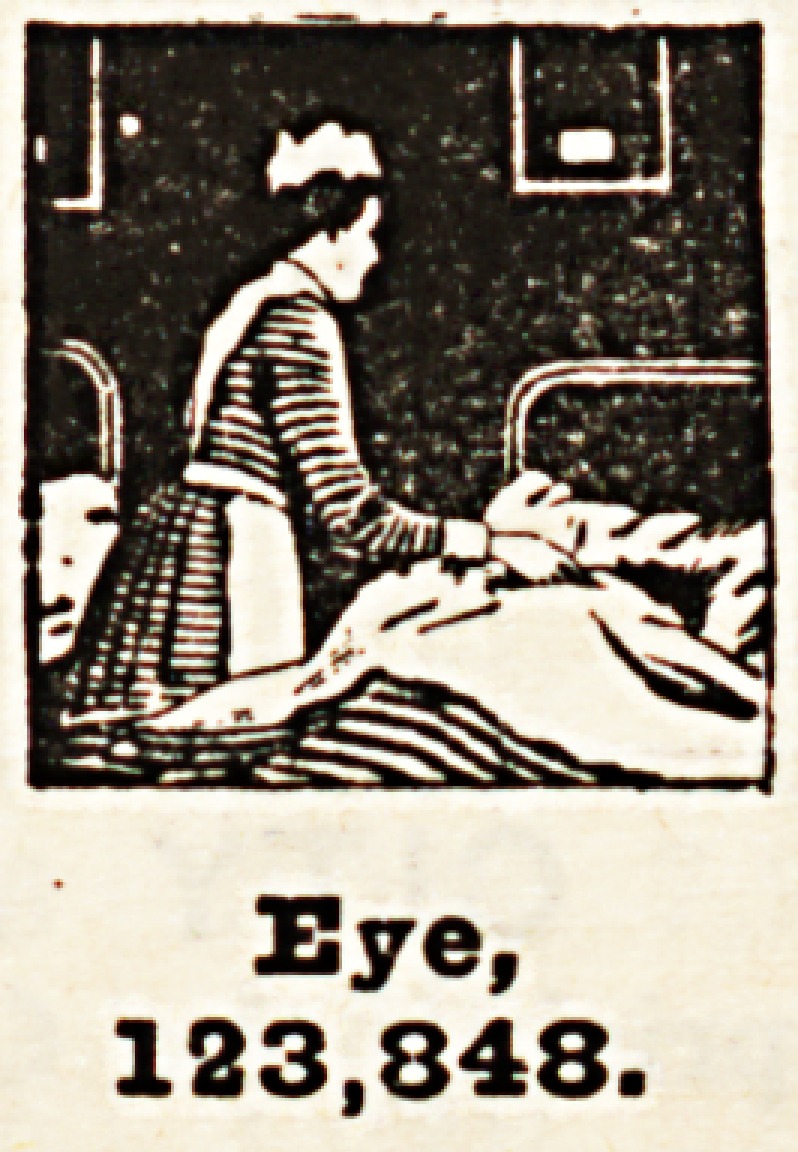


**Figure f12:**
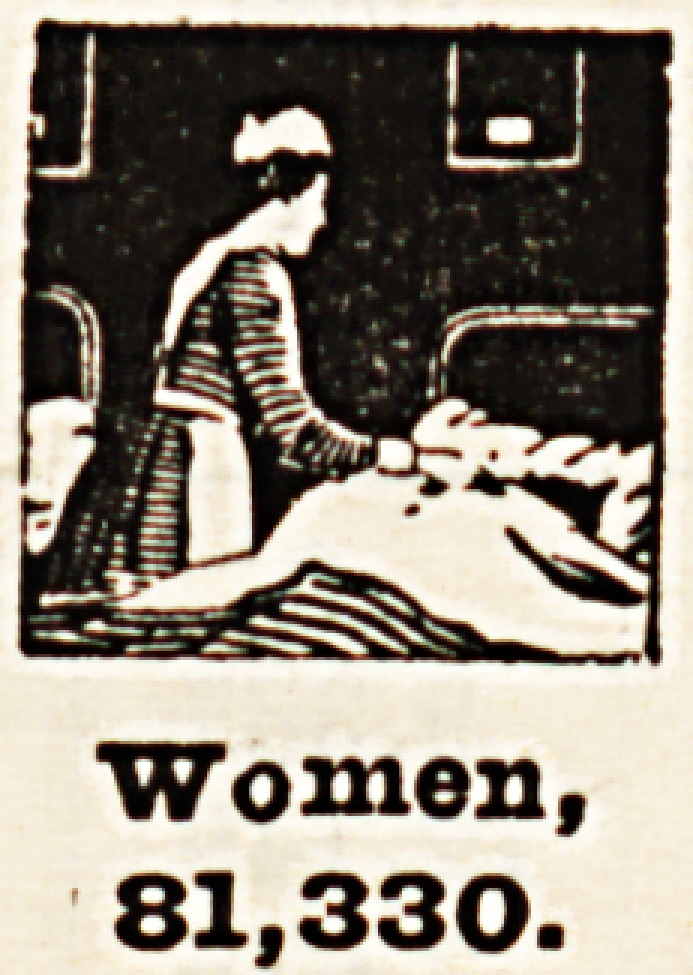


**Figure f13:**
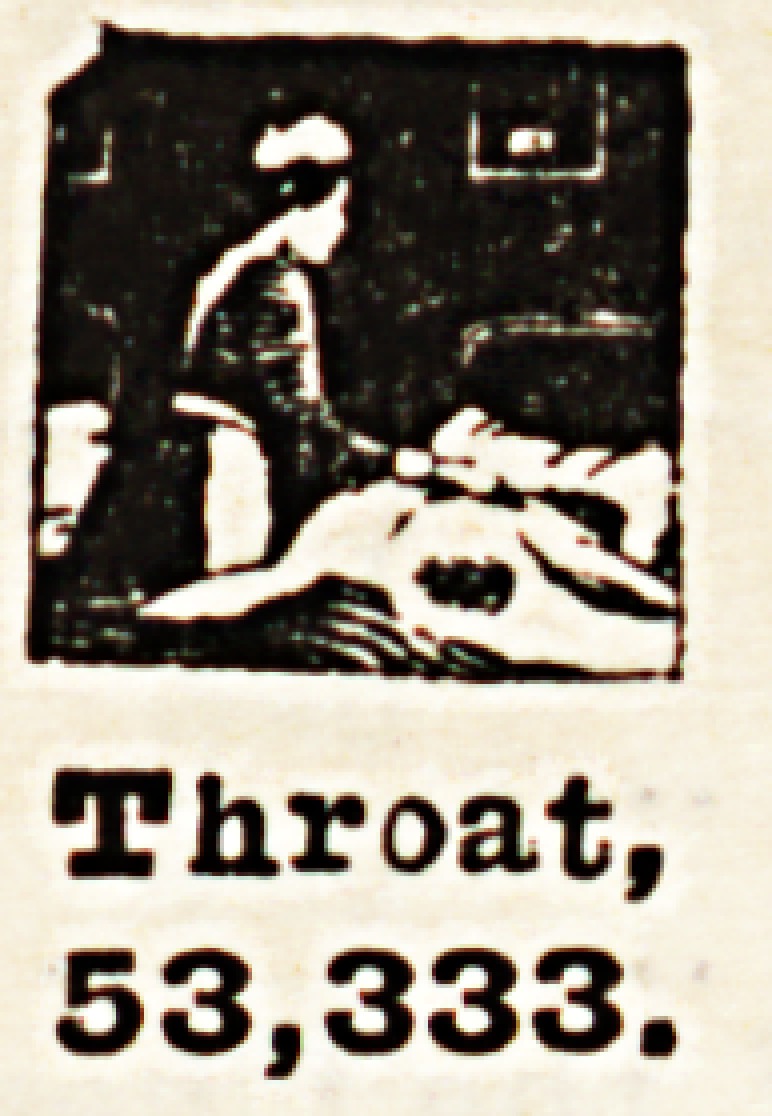


**Figure f14:**
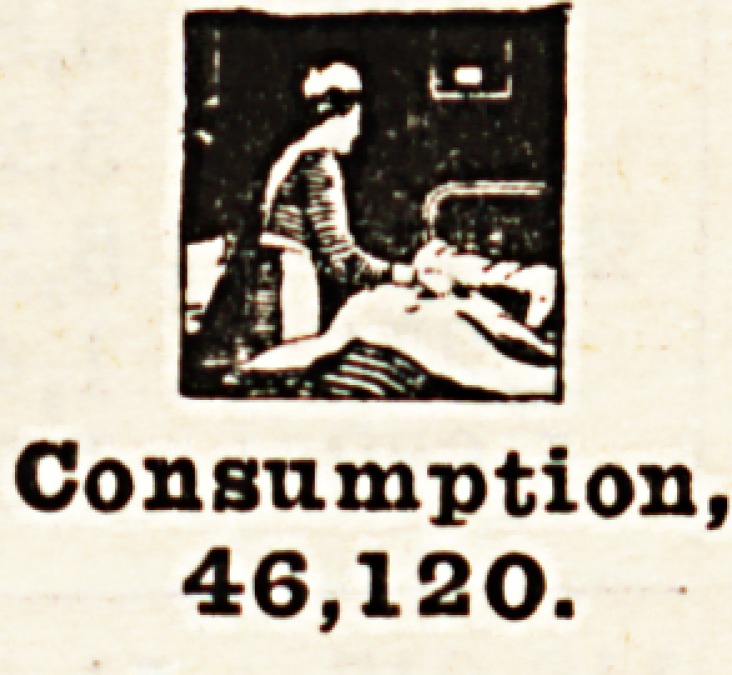


**Figure f15:**
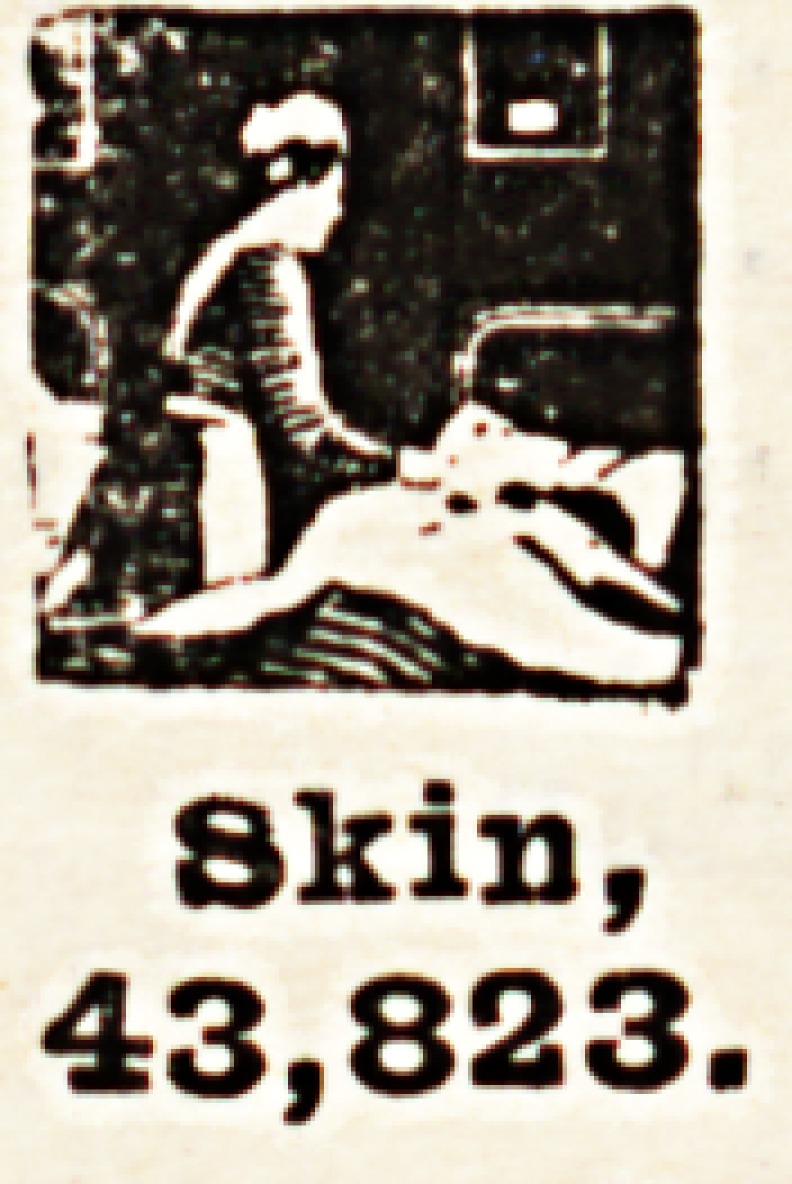


**Figure f16:**
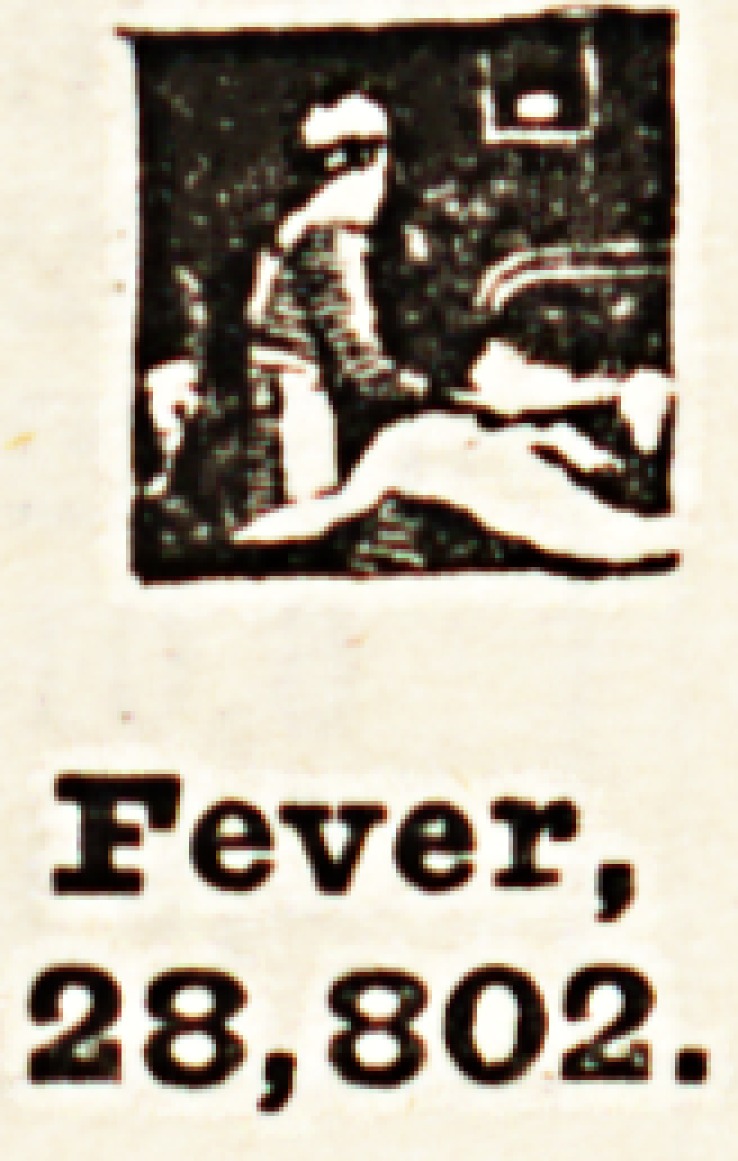


**Figure f17:**